# Effect of School-Based Physical Activity and Multi Micronutrient Supplementation on Micronutrient Concentrations Among Tanzanian Schoolchildren: Secondary Outcomes from the KaziAfya Cluster-Randomized Controlled Trial

**DOI:** 10.3390/nu18121980

**Published:** 2026-06-18

**Authors:** Elihaika G. Minja, Emmanuel C. Mrimi, Winfrida P. Mponzi, Johanna Beckmann, Marceline F. Finda, Fredros O. Okumu, Christin Lang, Markus Gerber, Jürg Utzinger, Kurt Z. Long

**Affiliations:** 1 Department of Environmental Health and Ecological Sciences, Ifakara Health Institute, Ifakara P.O. Box 53, Tanzania; emrimi@ihi.or.tz (E.C.M.); wmponzi@ihi.or.tz (W.P.M.); lfinda@ihi.or.tz (M.F.F.); fredros@ihi.or.tz (F.O.O.); 2 Swiss Tropical and Public Health Institute, Kreuzstrasse 2, CH-4123 Allschwil, Switzerland; juerg.utzinger@swisstph.ch (J.U.); kurt.long@swisstph.ch (K.Z.L.); 3 Faculty of Medicine, University of Basel, Petersplatz 1, CH-4003 Basel, Switzerland; 4 School of Public Health, Faculty of Health Sciences, University of the Witwatersrand, Johannesburg 2050, South Africa; markus.gerber@unibas.ch; 5 Department of Sport, Exercise and Health, University of Basel, Grosse Allee 6, CH-4052 Basel, Switzerland; johanna.beckmann@unibas.ch (J.B.); christin.lang@unibas.ch (C.L.); 6 School of Life Science and Bioengineering, Nelson Mandela African Institution of Science & Technology, Arusha P.O. Box 447, Tanzania; 7 School of Biodiversity, One Health and Veterinary Medicine, University of Glasgow, Glasgow G12 8QQ, UK

**Keywords:** multi-micronutrient supplementation, physical activity, school children, randomized controlled trial, micronutrients status

## Abstract

**Background:** Micronutrient deficiencies and physical inactivity can adversely affect child growth and development. This study assessed the effects of school-based physical activity and multi-micronutrient supplementation on micronutrient status among schoolchildren in Kilombero district, Tanzania. **Methods:** In a cluster-randomized trial, children aged 6–12 years were allocated to physical activity, multi-micronutrient supplementation, combined physical activity plus supplementation, or placebo control. Anthropometric and biochemical assessments were conducted at baseline, 14 months, and 26 months. Dried blood spot samples were available for 923 children at baseline. Complete-case analyses used biomarker-specific subsamples with valid baseline and 26-month measurements. **Results:** The primary complete-case sample included 243 children with valid paired measurements for zinc and serum transferrin receptor; vitamin D analyses were restricted to 52 children because of missing or invalid samples. At baseline, iron and vitamin D deficiencies were common, affecting 42.8% and 39.9% of children, respectively, while zinc deficiency affected 11.9%. At 26 months, allocation to the physical activity intervention was associated with lower odds of zinc deficiency, both when delivered alone (OR = 0.16) and when combined with supplementation (OR = 0.57). Supplementation alone was not significantly associated with reduced zinc deficiency. Iron status did not differ between intervention groups. Vitamin D findings should be interpreted with caution because analyses were based on a very small biomarker-specific subsample. **Conclusions:** School-based physical activity, alone or combined with multi-micronutrient supplementation, was associated with lower odds of zinc deficiency among Tanzanian schoolchildren. Supplementation alone showed no clear benefit for zinc or iron status. Vitamin D findings remain inconclusive because of substantial biomarker-specific missingness. Future trials should strengthen adherence monitoring, biomarker follow-up, and repeated assessment of dietary and contextual factors.

## 1. Introduction

Micronutrient deficiencies remain an important contributor to the global burden of disease and are associated with increased morbidity and mortality, particularly among vulnerable populations [1,2]. Children and pregnant women are disproportionately affected because of their increased nutritional requirements and greater susceptibility to the consequences of inadequate intake [2,3]. Deficiencies of iron, vitamin A, iodine, folate, and zinc are among the most common and may impair growth, immune function, cognitive development, and overall health [4]. Despite global efforts to improve nutrition, micronutrient deficiencies remain widespread in low- and middle-income countries, especially in sub-Saharan Africa, where dietary diversity is often limited and access to fortified foods or supplements may be inadequate [5].

In these settings, poor micronutrient status is usually caused by several overlapping factors, including low dietary diversity, recurrent infections, inflammation, poor nutrient bioavailability from predominantly plant-based diets, and limited access to health and nutrition services [5]. Among school-aged children, micronutrient deficiencies may increase vulnerability to illness and contribute to school absenteeism [6,7]. They may also have longer-term consequences for physical growth, learning, cognitive development, and future productivity In addition, some micronutrient deficiencies, including vitamin D, iron, and selected trace elements, may interact with emerging cardiometabolic risks, although these relationships vary by nutrient, population, and context [8,9].

Physical activity (PA) is also an important determinant of child health. Regular PA supports cardiovascular fitness, healthy body composition, sleep, mental health, and cognitive function [10,11]. However, PA levels among children and adolescents have declined in many settings [12,13]. For example, longitudinal evidence shows that total PA declines from childhood into adolescence [12,13], while global estimates indicate that most adolescents do not meet recommended levels of moderate-to-vigorous PA [14]. Childhood physical activity can help children stay active later in life [15]. school-based strategies are important for promoting active lifestyles and preventing long-term sedentary behavior [15]. Evidence from systematic reviews also suggests that school-based PA programs can improve PA and fitness among children and adolescents aged 6–18 years [16].

Previous studies have shown that single or multiple micronutrient interventions can improve vitamin A [17,18,19,20], zinc [17,21], and iron status [17,22], and may reduce anemia among school-aged children [18,20,21]. However, most studies have evaluated PA and micronutrient supplementation separately. Evidence regarding the combined effect of school-based PA and multi-micronutrient supplementation (MMNS) on micronutrient status remains scarce, particularly among school-aged children in resource-limited settings such as rural Tanzania.

Against this background, we hypothesized that a school-based intervention combining enhanced PA opportunities and MMNS would improve micronutrient status among school-aged children in Kilombero district, south-eastern Tanzania. This cluster-randomized controlled trial therefore assessed the effects of PA, MMNS, and their combination on selected micronutrient biomarkers among Tanzanian schoolchildren.

## 2. Methods

### 2.1. Ethics Statement

Ethical approval was obtained from the relevant local authorities, the Institutional Review Board of the Ifakara Health Institute (IHI; reference no. IHI/IRB/no. 39-2018, 20 December 2018), the Medical Research Coordinating Committee (MRCC) of the National Institute for Medical Research (NIMR; reference no. NIMR/HQ/R.8a/Vol. IX/3137, 17 June 2019), and the Ethics Commission of Northwestern and Central Switzerland (EKNZ; reference no. Req-2018-00608, 13 August 2018). Caregivers attended school-based information sessions, during which the study aims, procedures, potential risks, and benefits were explained. Written informed consent was obtained from caregivers. For caregivers who were unable to read or write, consent was documented using a thumbprint in the presence of a literate witness. On the following day, children whose caregivers had provided consent received an explanation of the study. Oral assent was obtained from all participating children after their questions had been addressed.

### 2.2. Recruitment Procedures

Eligible schools were selected based on predefined criteria, and school heads were informed about the study objectives and procedures. Caregivers of eligible children attended informational sessions and provided written or witnessed thumbprint consent. Children aged 6–12 years enrolled in grades 1–4 were eligible provided they had no medical conditions that would restrict participation in regular PA. Following parental consent, children were briefed and invited to give oral assent. Recruitment procedures and criteria have been described in detail elsewhere [23].

### 2.3. Study Design

This study utilized longitudinal data collected from Tanzanian schoolchildren between 2019 and 2021 as part of the KaziAfya project, a four-arm cluster-randomized controlled trial employing a 2 × 2 factorial design to evaluate the effects of PA, MMNS, or their combination (PA + MMNS) on growth, health, and well-being outcomes. KaziAfya is a multicounty trial conducted in Tanzania, Côte d’Ivoire, and South Africa [23]. Participants were monitored throughout two academic years, with data collection conducted at baseline (T1), 14 months post-baseline (T2), and 26 months post-baseline (T3). The clinical trial registration code is ISRCTN29534081. The study was registered with ISRCTN on 9 August 2018 and can be accessed here: https://doi.org/10.1186/ISRCTN29534081.

### 2.4. Intervention

A total of 1034 schoolchildren were enrolled and participated in the study. Children were assigned unique identification numbers after obtaining parent/guardian written informed consent and children’s oral assent. Classes within each school underwent random allocation to one of four study arms: PA, MMNS, PA + MMNS, or placebo control condition. Randomization methods allowed equal distribution to the three intervention arms and the control group across the four grades (grade 1–4) at each school.

The PA intervention commenced in February 2020 but was interrupted by a 6-month school closure due to COVID-19 pandemic restriction measures, resuming from August 2020 through November 2021. MMNS distribution to participating schoolchildren was initiated in February 2021 and continued until November 2021.

### 2.5. Physical Activity

Children assigned to the PA or PA + MMNS groups received two 45 min PA classes per week. Children assigned to physical activity or combined intervention groups receive two 45 min physical activity classes per week. These classes encompassed activities such as “moving to music” and “physical education”. Project staff who were physical education instructors who trained in implementing KaziKidz-lesson plans (www.kazibantu.org) provided guidance to classroom teachers during the moving-to-music and physical education lessons to ensure effective intervention implementation. The teaching materials were only given to teachers of the PA intervention classes. Children allocated to MMNS alone or the placebo control group received the usual school lessons.

### 2.6. Multi-Micronutrient Supplementation

Children assigned to the MMNS-alone or PA + MMNS groups received one daily chewable tablet containing vitamins and trace elements. The tablets were administered by class teachers in the morning on school days. The supplement was based on the MixMe™ powder sprinkle formulation provided by DSM Nutritional Products, Basel, Switzerland (Table 1). In this formulation, vitamin A was replaced by β-carotene, with each tablet containing 3.6 mg β-carotene.

The MMNS were manufactured, packaged, stored, and distributed in accordance with established quality-control procedures, and undergo quality checks before delivered to the schools. Teachers administered the MMNS and placebo tablets similar in taste and appearance five days per week to the students in the different intervention arms.

### 2.7. Data and Sample Collection

Details on the procedures of the entire study, including anthropometric measurements of schoolchildren and collection of all demographics, socioeconomic status (SES), food insecurity, and dietary diversity data were presented in our previous publication [24]. In brief, body mass index (BMI) was calculated as weight in kilograms divided by height in meters squared (kg/m^2^). Dietary diversity was assessed via a food frequency questionnaire (FFQ), a 24 h dietary recall adapted from United Nations’ Food and Agriculture Organization (FAO) administered to the caregivers [25]. It was adapted to reflect food names and items that were common in Tanzania and the study site. Full data collection procedures for the assessment of dietary diversity are described in detail in Minja et al. [24]. Dietary diversity was classified as low dietary diversity (scores ≤3, LDD), medium dietary diversity (scores 4–6, minimum dietary diversity (MDD)), and high dietary diversity (scores ≥7, high dietary diversity (HDD)), with the latter indicating food security [25]. The calculation procedure for dietary diversity has been described in detail by Minja et al. [24].

To assess food insecurity, we used the household hunger scale (HHS), a cross-culturally validated tool [26] that allows for direct comparison across different settings. In the present study, we used three standardized items; namely (i) “Was there ever no food to eat of any kind in your house because of lack of resources to obtain food?”; (ii) “Did you or any household member go to sleep at night hungry because there was not enough food?”; and (iii) “Did you or any household member go a whole day and night without eating anything because there was not enough food?” Each item was scored on a 3-point scale: 0 (never), 1 (rarely or sometimes), and 2 (often). Responses were aggregated to derive a household hunger score ranging from 0 to 6, with higher values denoting greater severity of food insecurity. Classification of household food insecurity was based on established thresholds [27], as described in detail elsewhere [28].

For micronutrient analysis, blood samples were collected from children at T1, T2, and T3. Five 50 µL volumes of blood were drawn into 0.5 mL EDTA microtainer tubes and subsequently spotted onto dried blood spot (DBS) cards. The DBS cards were air-dried completely, packaged with silica gel desiccants, and stored at sub-zero temperatures until analysis. The DBS samples were then shipped to the Global Clinical and Viral Laboratory in Durban, South Africa.

Eluted dry blood spots were screened for retinol binding protein (RBP), a marker of vitamin A, vitamin D, zinc, and serum transferrin receptor (sTfR), the latter a marker of iron deficiency. Analyses were done at the Neuberg Global Laboratories in South Africa. Briefly, sandwich-ELISA commercial kits for sTfR (Elabscience; Houston, TX, USA), vitamin D (Euroimmun; Lübeck, Germany), and RBP (Arbor Assays; Ann Arbor, MI, USA) were used with samples added to micro plate wells precoated with antibodies for the specific marker followed by addition of biotinylated detection antibody and Avidin-HRP conjugate. For the zinc assay (Biorex Diagnostics; Antrim, UK), zinc present in the sample was chelated with 5-BR-PAPS and then read. Micronutrient deficiencies were defined using established clinical cut-offs. Vitamin A was categorized into two groups: adequate (>0.2 g/mL) and deficient (<0.2 g/mL) [29]. For vitamin D, samples with >20 ng/mL were considered adequate, while those with <20 ng/mL were considered deficient [30]. For sTfR: <8.3 mg/L (adequate iron), >8.3 mg/L (iron deficiency) [31], and zinc concentrations <10.7 µmol/L were considered deficient, while concentrations ≥10.7 µmol/L were considered adequate [32]. Inflammation markers, such as C-reactive protein (CRP) and α1-acid glycoprotein (AGP), were not available for the current secondary analysis. Therefore, micronutrient biomarker concentrations could not be adjusted for inflammation.

### 2.8. Statistical Analysis

This was a secondary analysis of micronutrient biomarker outcomes from the KaziAfya cluster-randomized controlled trial. No separate sample-size calculation was conducted for the biomarker outcomes; analyses were therefore restricted to children with valid baseline and 26-month biomarker and covariate data. The 14-month assessment was not analyzed because multi-micronutrient supplementation had not yet started and physical activity exposure had been limited by COVID-19-related school closures. Descriptive statistics are presented as means with 95% confidence intervals or frequencies and percentages. Baseline differences by sex were assessed using *t*-tests or chi-square tests, as appropriate. Follow-up differences between intervention groups were assessed using analysis of variance or chi-square tests.

Intervention effects on micronutrient deficiency at 26 months were estimated using generalized estimating equation logistic models with school-level clustering, an exchangeable working correlation structure, and robust standard errors. The placebo group was used as a reference. Adjusted models included sex, baseline age, baseline biomarker concentration, dietary diversity, and household food security. Additional factorial models included physical activity, supplementation, and their interaction term.

The analysis followed an allocation based complete-case approach. Missing biomarker outcomes were not imputed because most missingness resulted from specimen loss, degradation, insufficient dried blood spot material, or assay-specific failure. Findings are therefore interpreted cautiously, particularly for vitamin D, which had the smallest biomarker-specific complete-case sample.

## 3. Results

### 3.1. Baseline Assessment

**Figure 1:**
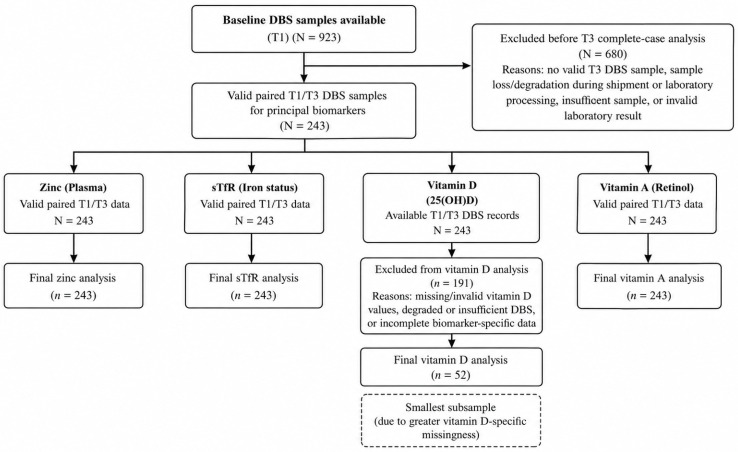
Participant flow diagram showing biomarker-specific inclusion and exclusion from baseline DBS collection to the T3 complete case analytic samples. Exclusions were mainly due to missing or invalid follow-up DBS measurements, incomplete covariate information, sample loss, degradation during storage, shipment, or laboratory processing, and biomarker-specific invalid laboratory results. Final analytic samples differed by biomarker because complete-case analyses were conducted separately for each outcome.

At baseline, DBS samples were successfully collected and analyzed for micronutrient concentrations from 923 schoolchildren. After exclusions due to missing or invalid follow-up DBS samples, sample loss, degradation during shipment or laboratory processing, and incomplete covariate information, 243 children had valid paired T1/T3 DBS measurements for the principal biomarkers. These 243 children constituted the primary complete-case sample used in Table 2 and in the zinc and sTfR models, while vitamin D analyses used a smaller biomarker-specific complete-case subsample of 52 children. The revised participant flow diagram explicitly shows biomarker-specific exclusions and summarizes the main reasons for missingness, including missing or invalid T3 DBS measurements, incomplete covariate information, and sample loss or degradation (Figure 1).

Among the 243 children in the primary complete-case sample, the mean age at baseline was 8.6 years (95% CI: 7.7–9.5 years, Table 2). No significant differences were observed between boys and girls in mean age (*p* = 0.11), height (*p* = 0.10), or weight (*p* = 0.23). No significant sex difference was observed in BMI at baseline (*t* = −0.01, *p* = 0.988).

Regarding micronutrient status, 39.9% of children were vitamin D deficient, with no significant sex differences (*p* = 0.22). Iron deficiency, based on elevated sTFR, was observed in 42.8% of the sample, with comparable prevalence between boys and girls (*p* = 0.68). Zinc deficiency was observed in 29 (11.9%) children with no sex difference (*p* = 0.94, Table 2).

To address potential confusion about denominators, Table 3 reconciles the sample sizes used at each stage of analysis. The baseline DBS denominator (*n* = 923) reflects sample availability at T1, whereas the primary complete-case denominator (*n* = 243) reflects children with valid paired T1/T3 DBS data for the main biomarker analyses. The vitamin D denominator is smaller (*n* = 52) because of missing or invalid vitamin D measurements.

### 3.2. Post-Intervention Assessment

In the primary complete-case analytic sample (*N* = 243) at T3, no statistically significant differences were observed between groups for age, height, weight, BMI, or sTFR status (all *p* > 0.05). However, sex distribution differed between groups (*p* < 0.01), with a higher pro-portion of girls in PA (75%) and a lower proportion in MMNS (43%), reflecting the composition of the restricted analytic sample. Differences between intervention groups were observed for selected micronutrient outcomes. Vitamin D results were based on the smaller biomarker-specific subsample (*n* = 52), with vitamin D deficiency being most prevalent in the placebo group (45%) and not being observed in the MMNS or PA + MMNS groups. Zinc differed significantly (*p* < 0.01), with zinc deficiency being most prevalent in the placebo group (23%) and lowest in the PA group (5%), while sTFR did not differ (*p* = 0.95) (Table 4).

In the GEE analysis, the odds of zinc deficiency were significantly lower in the PA group compared with the control group and were also lower in the PA + MMNS group, while no significant difference was observed for MMNS alone (Table 5). After controlling for covariates, the associations remained consistent: lower odds of zinc deficiency in PA (OR = 0.16, 95% CI 0.06, 0.42) and PA + MMNS (OR = 0.57, 95% CI 0.37, 0.86), with no significant effect for MMNS alone. Baseline age was positively associated with the odds of zinc deficiency. The zinc and sTfR models were based on the primary complete-case sample (*n* = 243), whereas the vitamin D model was based on the vitamin D complete-case subsample (*n* = 52).

### 3.3. Vitamin D

There were no differences in vitamin D deficiency among children in the different intervention groups compared with the control group in the unadjusted models (Table 5). After adjustment for covariates, MMNS (OR = 1.73, 95% CI 1.40, 2.15) and PA + MMNS (OR = 2.56, 95% CI 1.45, 4.54) were associated with higher odds of vitamin D deficiency compared with control, while PA showed no difference. These estimates should be interpreted cautiously because the vitamin D model was based on the smallest biomarker-specific complete case subsample (*n* = 52). Sex was protective, with females having lower odds of deficiency (OR = 0.60, 95% CI 0.50, 0.72). Baseline age was associated with higher odds of deficiency (OR = 1.20, 95% CI 1.02, 1.42). Higher baseline concentrations were linked to reduced odds (OR = 0.89, 95% CI 0.85, 0.95). Among dietary and food security indicators, higher household dietary diversity (HDD) was unexpectedly associated with greater odds of deficiency (OR = 2.58, 95% CI 1.24, 5.34). Moderate hunger was associated with increased odds (OR = 1.79, 95% CI 1.28, 2.52), whereas severe hunger was linked to reduced odds (OR = 0.66, 95% CI 0.50, 0.86).

### 3.4. sTfR

No significant differences were observed between intervention groups and control. After covariate adjustment, baseline age was positively associated with higher odds of elevated sTfR. Higher baseline concentrations were linked to lower odds Among dietary and food-security indicators, medium dietary diversity and high dietary diversity were associated with lower odds of elevated sTfR. Associations with hunger categories were not significant.

## 4. Discussion

This study examined the effects of PA, MMNS, and their combination on micronutrient concentrations among children aged 6–12 years in Kilombero district. The baseline DBS sample was large (*n* = 923), but valid paired T1 and T3 DBS/laboratory data were available for a smaller primary complete-case sample (*n* = 243), while vitamin D analyses were based on an even smaller subsample (*n* = 52). Because the complete-case analyses were based on biomarker-specific subsamples, selection bias due to missing or invalid biomarker data cannot be ruled out. This limitation is particularly important for vitamin D, which had the smallest analytic sample. Therefore, the intervention estimates should be interpreted as complete-case findings, with caution for outcomes affected by substantial biomarker-specific missingness. The interventions demonstrated heterogeneous effects, with notable improvements observed for zinc status in the PA and PA + MMNS groups, but no clear benefits for vitamin D or iron status. These findings should also be interpreted in the context of staggered intervention timing and COVID-19-related disruption. Because PA and MMNS were not delivered over identical periods, and school closures reduced continuity of implementation, the observed differences may reflect both intervention allocation and variation in actual exposure.

### 4.1. Zinc

Previous studies examining the relationship between PA and zinc status have produced mixed findings. Cross-sectional studies and reviews comparing athletes or physically active groups with less active controls have reported variable results, including lower serum zinc concentrations among athletes despite higher dietary zinc intake, but the evidence for long-term exercise effects on zinc status remains incomplete [33,34]. In the present study, allocation to the PA group, either alone or in combination with multi-micronutrient supplementation, was associated with lower odds of zinc deficiency at follow-up. However, the mechanisms underlying this association cannot be established from the present data.

Several biological and contextual pathways may be hypothesized, but they should be interpreted cautiously. PA could potentially influence appetite, total food intake, gastrointestinal function, immune regulation, or infection-related zinc losses [35,36,37,38]. However, changes in appetite, dietary intake during the intervention period, zinc absorption, inflammation, and infection burden were not directly measured in this study. Therefore, these explanations remain speculative. The observed association may also reflect unmeasured differences in dietary intake, adherence, infection exposure, inflammation, or other contextual factors. Thus, while the zinc finding is noteworthy, it should be interpreted as an association between PA allocation and zinc status rather than definitive evidence that PA directly improved zinc absorption or metabolism.

The lack of a significant association between MMNS alone and reduced zinc deficiency should also be interpreted cautiously. Previous studies have shown that MMNS can improve plasma or serum concentrations of ferritin, retinol, and zinc and reduce zinc deficiency among school-aged children [17,39]. In the present setting, however, several contextual factors may have limited the detectable effect of supplementation. The predominantly plant-based diets in the study population are likely to contain phytates from cereals and legumes, which can inhibit zinc absorption [40,41,42]. The zinc dose provided in the supplement may therefore have been insufficient to overcome phytate-related limitations in bioavailability. In addition, mineral interactions within multi micronutrient formulations, particularly between zinc and other divalent minerals such as iron and calcium, may influence absorption under some conditions [43]. Finally, the shortened and disrupted intervention period may have limited the ability to detect measurable improvements in zinc status, especially because zinc has limited specialized body stores and requires regular dietary intake to maintain adequate status [42,44].

Overall, the findings suggest a possible beneficial association between PA allocation and zinc status, but they do not identify the biological pathway responsible for this association. Future studies should include repeated dietary intake assessments, supplement adherence measures, inflammation markers, infection data, and, where feasible, biomarkers of zinc absorption or metabolism to clarify whether changes in zinc status are mediated by diet, absorption, inflammation, or other mechanisms.

### 4.2. Vitamin D

The vitamin D analyses should be interpreted with caution because they were based on the smallest biomarker specific complete case subsample (*n* = 52). This was due to additional missing or invalid 25-hydroxyvitamin D [25(OH)D] measurements beyond the general loss of paired dried blood spot samples. The small sample size reduced statistical power, widened uncertainty around the estimates, and increased the possibility of selection bias. Although baseline vitamin D concentrations and age were broadly comparable between included and not included children, sex imbalance was observed, suggesting that the analytic subsample may not have fully preserved the balance expected from the original randomized cohort.

The vitamin D results were also inconsistent across analytic approaches. In the unadjusted T3 comparison, vitamin D deficiency appeared lower in the MMNS and PA + MMNS groups than in the control group. However, in the adjusted endpoint GEE models, which accounted for baseline vitamin D concentration and other covariates, MMNS and PA + MMNS were associated with higher odds of vitamin D deficiency. These differences likely reflect the small and selective analytic sample, differential missingness, and residual confounding rather than clear evidence of a true adverse intervention effect.

Seasonal variation in sunlight exposure and timing of blood collection may also have influenced vitamin D concentrations [45]. In addition, adherence to MMNS and participation in PA sessions may have varied during the disrupted intervention period, which could have weakened or distorted intervention effects [46]. Overall, the present findings do not provide consistent evidence that MMNS, either alone or combined with PA, improved vitamin D status. Given the small sample size and potential selection bias, the vitamin D findings should be considered exploratory and require confirmation in studies with more complete vitamin D measurements and better control for seasonality.

### 4.3. sTfR

Neither PA nor MMNS improved iron status as assessed by sTfR. This may reflect limited intervention intensity and duration, combined with contextual constraints on iron absorption and utilization. Although MMNS provided iron, the dose and/or nutrient interactions (e.g., with calcium or zinc) may have reduced net absorption [43]. In addition, the predominantly plant based diet in the study area is high in phytates, which strongly inhibit non-heme iron bioavailability [47]. Finally, infections and inflammation common in the study area can limit the effectiveness of iron interventions by altering iron metabolism and reducing utilization [48,49], promote iron sequestration and impair utilization, thereby blunting the impact of supplementation [50]. Together, these factors likely reduced the potential for measurable improvement in iron status during the study period.

### 4.4. Strengths and Limitations

A key strength of this study is its focus on school-aged children, a population group particularly responsive to targeted nutritional and behavioral interventions. In addition, the study was embedded within a larger multi-country cluster-randomized controlled trial, conducted under a standardized protocol and procedures that were reviewed and approved across multiple institutional and ethical bodies. These features enhance both the rigor and external validity of the findings. Despite its unique contributions, this study has several limitations that should be considered.

First, intervention timing and exposure were not fully consistent. The PA intervention began before MMNS, but its delivery was interrupted by COVID-19-related school closures. MMNS started later and was delivered over a shorter period. This disrupted implementation complicates the interpretation of the 2 × 2 factorial design because the PA, MMNS, and PA + MMNS groups did not receive fully comparable intervention exposure. Therefore, the findings should be interpreted as allocation-based estimates under real world disrupted implementation conditions, rather than as definitive causal estimates of the independent and combined biological effects of PA and MMNS. COVID-19-related interruptions may also have reduced adherence, diluted intervention effects, and limited the ability to detect true differences between groups.

Second, selection bias due to biomarker specific missing data cannot be ruled out. Although 923 children had DBS samples at baseline, the final complete case analytic samples were substantially smaller because of missing T3 samples, sample loss or degradation, insufficient DBS material, invalid laboratory results, assay specific failure, and missing covariate data. Vitamin D was most affected, with the analytic sample reduced to *n* = 52. Since these losses may not have occurred completely at random, the included children may not fully represent the original randomized cohort. Baseline comparisons showed that age and most biomarker concentrations were broadly similar between included and not-included children, but sex imbalance was observed. This may have influenced the estimates despite adjustment for sex in the GEE models. Therefore, residual selection bias and reduced internal validity cannot be ruled out, particularly for vitamin D.

Third, the analysis was based on complete cases data set. Multiple imputation was considered but not performed because most missing outcome data arose from biological specimen loss, degradation, insufficient DBS material, or assay failure. Under these conditions, imputed T3 biomarker values would depend on strong assumptions that could not be verified from the available data. Similarly, inverse probability weighting was not performed. Future analyses with more complete laboratory recovery could consider multiple imputations or inverse probability weighting as formal sensitivity analyses.

Fourth, dietary diversity, household food security, and socioeconomic conditions were assessed at baseline but not repeatedly measured during follow-up. This is important because the study overlapped with the COVID-19 pandemic, during which household income, food access, market availability, school attendance, and dietary intake may have changed. Although baseline dietary diversity and household hunger were adjusted for in the models, changes in these factors over time could not be captured. Therefore, residual confounding by pandemic related socioeconomic and dietary changes cannot be ruled out.

Fifth, inflammation markers, such as C-reactive protein and α1-acid glycoprotein, were not available. Inflammation and infection can influence micronutrient biomarker concentrations, particularly iron-related markers, and may also affect zinc and vitamin A status. Therefore, residual bias due to unmeasured inflammation cannot be ruled out.

Finally, individual level adherence to MMNS intake and PA participation was not fully captured in the current secondary analysis. Although MMNS and placebo tablets were administered by teachers during school days, adherence may have varied because implementation occurred under COVID-19 related school closures and other pandemic-related disruptions. These interruptions may have led to deviations from the intended protocol and reduced actual intervention exposure. In addition, vitamin D concentrations may have been influenced by the timing or season of blood collection because sunlight exposure varies across months. Since complete individual level sampling date information was not available for all participants, adjustment for blood collection timing was limited, and residual confounding by seasonality cannot be ruled out. These limitations may have influenced the observed intervention effects, particularly for vitamin D.

## 5. Conclusions

In this secondary analysis, school-based physical activity, alone or combined with supplementation, was associated with lower odds of zinc deficiency at 26 months. Supplementation alone showed no clear benefit for zinc status, and none of the interventions improved iron status. Vitamin D findings remain inconclusive because of substantial biomarker-specific missingness and the small complete-case subsample. Future school-based nutrition trials should strengthen biomarker follow-up, adherence monitoring, and repeated assessment of diet, inflammation, and contextual factors that may affect micronutrient outcomes.

## Figures and Tables

**Table 1:** Content of the multi-micronutrient supplementation (MMNS) chewing tablets.

No.	Nutrient	Average Per Tablet
1	β-carotene (as BetTab 20%S)	3.6 mg
2	Vitamin D	400 IU/10 mcg
3	Vitamin E	9 mg TE
4	Vitamin K	30 mcg
5	Vitamin C	60 mg
6	Vitamin B1 thiamine	1.1 mg
7	Vitamin B2 riboflavin	1.3 mg
8	Vitamin B6 pyriodoxine	0.5 mg
9	Vitamin B12	1.2 mcg
10	Folic acid	200 mcg
11	Niacinamide	8 mg
12	Iron (added as Fe-EDTA)	8 mg
13	Zinc (added as zinc oxide)	5 mg
14	Selenium (added as sodium selenite anhydrous)	20 mcg
15	Iodine (added as potassium iodate)	100 mcg

**Table 2:** Descriptive statistics at T1 and differences between boys and girls in the primary complete-case sample, based on χ^2^-tests (categorical variables) and *t*-tests (continuous variables).

Measures	*N*	All Children (*N* = 243)	Boys (*n* = 98)	Girls (*n* = 145)	*t-*Value χ^2^	*p*-Value
		M (95% CI)	M (95% CI)	M (95% CI)		
Age (years)	243	8.0 (7.7, 9.5)	9.3 (8.9, 9.6)	8.1 (6.6, 9.6)	−1.58	0.11
Height (cm)	231	126.7 (125.4, 128.1)	128.0 (125.9, 130.2)	125.8 (124.1, 127.5)	−1.62	0.10
Weight (kg)	231	25.2 (24.5, 25.9)	25.7 (24.6, 26.7)	24.8 (23.9, 25.7)	−1.19	0.23
BMI (kg/m^2^)	231	15.54 (15.31, 15.76)	15.4 (15.2, 15.8)	15.5 (15.2, 15.8)	−0.01	0.98
**Micronutrient status (%)**						
**Vitamin D**						
Adequate	243	146 (60.1%)	64 (43.8%)	82 (56.2%)	1.52	0.22
Deficient		97 (39.9%)	34 (35.1%)	63 (65.0%)		
**Zinc**						
Adequate	243	214 (88.1%)	87 (40.7%)	127 (59.4%)	0.00	0.94
Deficient		29 (11.9%)	11 (37.9%)	18 (62.1%)		
**Iron deficiency marker**						
**sTFR**						
Adequate	243	139 (57.2%)	54 (38.9%)	85 (61.2%)	0.17	0.68
Deficient		104 (42.8%)	44 (42.3%)	60 (57.7%)		
**Dietary diversity**						
Low	241	29 (12.0%)	11 (37.9%)	18 (62.1%)	4.00	0.10
Medium		168 (69.7%)	73 (43.5%)	95 (56.6%)		
High		44 (18.3%)	12 (27.3%)	32 (72.7%)		
**Household food security**						
Little or no hunger	202	165 (67.9%)	68 (41.2%)	97 (58.8%)	0.27	0.87
Moderate hunger		32 (13.2%)	13 (40.6%)	19 (59.4%)		
Severe hunger		46 (18.9%)	17 (40.0%)	5 (63.0%)		

**Notes:** Table 2 is based on the primary complete-case sample (*N* = 243; 98 boys and 145 girls). Sample sizes vary by row because of missing anthropometric, dietary, or household data; baseline vitamin D, zinc, and sTfR status are shown for *N* = 243.

**Table 3:** Reconciliation of sample-size denominators used in the manuscript.

Denominator/Stage	Number of Samples	Clarification
Children enrolled in the Tanzania KaziAfya trial	N = 1034	Trial enrolment denominator before biomarker availability was assessed.
Baseline DBS samples available at T1	*n* = 923	Baseline laboratory denominator; not the final analytic sample.
No valid paired T3 DBS sample	*n* = 680	Missing, lost, invalid, or degraded by follow-up, shipment, or laboratory processing.
Primary DBS complete-case sample	*n* = 243 (145 girls, 98 boys)	Used for the abstract, Table 2, zinc models, and sTfR models; Table 2 header corrected from *N* = 293.
Vitamin D complete-case subsample	*n* = 52	Biomarker-specific denominator due to missing or invalid vitamin D values; findings interpreted cautiously.

Note: N (Total sample) and *n* (subsample). Missingness categories were grouped because some specimens had overlapping or incompletely recorded reasons for non-inclusion. The revised table and Figure 1 were added to address reviewer concerns about biomarker-specific denominators, missingness, and possible selection bias.

**Table 4:** Descriptive statistics at T3 and differences between the intervention conditions and placebo control group, based on χ2-tests (categorical variables) and ANOVAs (continuous variables).

Child Characteristics	Interventions					
	Placebo M (95% CI)	MMNS M (95% CI)	PA + MMNS M (95% CI)	PA M (95% CI)	χ^2^/F Score	*p*-Value
Girls, *n* (%)	42 (60%)	26 (43%)	31 (61%)	46 (75%)	13.67	**<0.01**
Boys, *n* (%)	28 (40%)	35 (57%)	20 (39%)	15 (24%)		
Age (years)	10.9 (10.7, 11.1)	10.4 (10.3, 10.6)	11.3 (11.2, 11.5)	9.54 (7.76, 11.3)	0.65	0.58
Height (cm)	135 (134.0, 137.0)	132 (131.0, 134.0)	126 (120.0, 132.0)	126 (119.0, 132.0)	1.11	0.35
Weight (kg)	31.8 (30.7, 32.9)	29.8 (28.9, 30.7)	26.2 (22.9, 29.5)	27.2 (23.4, 31.0)	0.95	0.42
BMI (kg/m^2^)	17.1 (16.8, 17.4)	16.8 (16.6, 17.1)	16.9 (16.6, 17.1)	17.7 (17.4, 18.0)	1.97	0.12
**Micronutrient status (%)**						
**Vitamin D**						
Adequate	17 (55%)	1 (100%)	4 (100%)	14 (87%)	7.74	**0.05**
Deficient	14 (45%)	0 (0%)	0 (0%)	2 (12%)		
**Zinc**						
Adequate	54 (775)	57 (93%)	45 (88%)	58 (95%)	12.48	**<0.01**
Deficient	16 (23%)	4 (7%)	6 (12%)	3 (5%)		
**Iron deficiency marker**						
**sTFR**						
Adequate	40 (57%)	36 (59%)	29 (57%)	38 (62%)	0.36	0.95
Deficient	30 (43%)	25 (41%)	22 (43%)	23 (37%)		

Note: Table 4 uses the primary complete-case sample (*N* = 243) for sex, anthropometry, zinc, and sTfR outcomes. Vitamin D percentages are based on the biomarker-specific complete-case subsample (*n* = 52; placebo *n* = 31, multi-micronutrient supplement group *n* = 1, physical activity plus multi-micronutrient supplement group *n* = 4, physical activity group *n* = 16), as shown in Figure 1.

**Table 5:** Effect of intervention on micronutrients status among Tanzanian school-aged children at T3: GEE model results.

Micronutrient Status	Zinc Deficiency			Vitamin D Deficiency			sTFR		
	Coefficient	OR (95% CI)	*p*-Value	Coefficient	OR 95% CI	*p*-Value	Coefficient	OR 95% CI	*p*-Value
**Unadjusted**									
Control	-	-	-	-	-	-	-	-	-
MMNS	−1.41	0.24 (0.02, 2.56)	0.23	−0.27	0.77 (0.38, 1.53)	0.45	−0.11	0.89 (0.36, 2.25)	0.81
PA	−1.75	0.17 (0.04, 0.67)	**0.01**	0.15	1.16 (0.56, 2.43)	0.68	−0.21	0.81 (0.36, 1.82)	0.62
PA + MMNS	−0.70	0.49 (0.37, 0.67)	**<0.001**	−0.15	0.86 (0.35, 2.08)	0.74	−0.05	0.95 (0.28, 3.21)	0.93
**Adjusted**									
Control	-	-	-	-	-	-	-	-	-
MMNS	−1.39	0.25 (0.03, 1.77)	0.16	0.55	1.73 (1.40, 2.15)	**<0.001**	−0.02	0.97 (0.46, 2.05)	0.94
PA	−1.83	0.16 (0.06, 0.42)	**0.00**	−0.07	0.93 (0.57, 1.53)	0.78	−0.31	0.73 (0.41, 1.29)	0.28
PA + MMNS	−0.56	0.57 (0.37, 0.86)	**<0.001**	0.94	2.56 (1.45, 4.54)	**<0.001**	−0.19	0.82 (0.25, 2.74)	0.75
Sex	0.13	1.14 (0.62, 2.07)	0.67	−0.51	0.60 (0.50, 0.72)	**<0.001**	−0.04	0.95 (0.49, 1.83)	0.88
Age at baseline	0.16	1.12 (1.08, 1.26)	**<0.001**	0.18	1.20 (1.02, 1.42)	**0.02**	0.18	1.19 (1.06, 1.34)	**0.02**
Baseline concentration	−0.01	0.99 (0.91, 1.07)	0.79	−0.11	0.89 (0.85, 0.95)	**<0.001**	−0.53	0.59 (0.41, 0.84)	**<0.001**
MDD	0.61	1.84 (1.33, 2.56)	**<0.001**	0.64	1.89 (0.55, 6.57)	0.31	−0.84	0.43 (0.26, 0.71)	**<0.001**
HDD	−0.10	0.90 (0.33, 2.47)	0.84	0.95	2.58 (1.24, 5.34)	**0.01**	−0.59	0.55 (0.47, 0.64)	**<0.001**
Moderate hunger	0.83	2.29 (1.63, 3.22)	**<0.001**	0.58	1.79 (1.28, 2.52)	**<0.001**	0.03	1.00 (0.52, 1.93)	0.99
Severe hunger	0.30	1.35 (0.74, 2.45)	0.32	−0.42	0.66 (0.50, 0.86)	**<0.001**	−0.17	0.84 (0.42, 1.66)	0.62

**Notes:** PA = physical activity group; MMNS = multi-micronutrient supplement group; PA + MMNS = physical activity plus multi-micronutrient supplement group, DDS = dietary diversity score, HHS = household hunger scale. Models are adjusted for age, sex, base-line concentration, DDS, and HHS at T1. Zinc and sTfR models used the primary complete-case sample (*n* = 243); the vitamin D model used the complete-case subsample (*n* = 52). Statistically significant differences are marked with bold font. The control group is used as a reference.

## Data Availability

The original contributions presented in this study are included in the article. Further inquiries can be directed to the corresponding author.
